# The Cooperative Relationship between STAT5 and Reactive Oxygen Species in Leukemia: Mechanism and Therapeutic Potential

**DOI:** 10.3390/cancers10100359

**Published:** 2018-09-27

**Authors:** Tian Mi, Zhengqi Wang, Kevin D. Bunting

**Affiliations:** 1 Department of Pediatrics, Division of Hem/Onc/BMT and Aflac Cancer and Blood Disorders Center, Emory University School of Medicine, 1760 Haygood Dr. NE, HSRB E308, Atlanta, GA 30322, USA; tian.mi@emory.edu (T.M.); zhengqi.wang@emory.edu (Z.W.); 2 Emory Winship Cancer Institute, Atlanta, GA 30322, USA

**Keywords:** signal transducer and activator of transcription, STAT5, reactive oxygen species, leukemogenesis, molecular targeted drug therapy

## Abstract

Reactive oxygen species (ROS) are now recognized as important second messengers with roles in many aspects of signaling during leukemogenesis. They serve as critical cell signaling molecules that regulate the activity of various enzymes including tyrosine phosphatases. ROS can induce inactivation of tyrosine phosphatases, which counteract the effects of tyrosine kinases. ROS increase phosphorylation of many proteins including signal transducer and activator of transcription-5 (STAT5) via Janus kinases (JAKs). STAT5 is aberrantly activated through phosphorylation in many types of cancer and this constitutive activation is associated with cell survival, proliferation, and self-renewal. Such leukemic activation of STAT5 is rarely caused by mutation of the STAT5 gene itself but instead by overactive mutant receptors with tyrosine kinase activity as well as JAK, SRC family protein tyrosine kinases (SFKs), and Abelson murine leukemia viral oncogene homolog (ABL) kinases. Interestingly, STAT5 suppresses transcription of several genes encoding antioxidant enzymes while simultaneously enhancing transcription of NADPH oxidase. By doing so, STAT5 activation promotes an overall elevation of ROS level, which acts as a feed-forward loop, especially in high risk Fms-related tyrosine kinase 3 (FLT3) mutant leukemia. Therefore, efforts have been made recently to target ROS in cancer cells. Drugs that are able to either quench ROS production or inversely augment ROS-related signaling pathways both have potential as cancer therapies and may afford some selectivity by activating feedback inhibition of the ROS-STAT5 kinome. This review summarizes the cooperative relationship between ROS and STAT5 and explores the pros and cons of emerging ROS-targeting therapies that are selective for leukemia characterized by persistent STAT5 phosphorylation.

## 1. Introduction

Reactive oxygen species (ROS) are cellular molecules that are generated primarily as a by-product of mitochondrial oxidative metabolism or NADPH oxidase enzymes [1]. ROS consist of radical and non-radical oxygen species including superoxide anion (O_2_−), hydrogen peroxide (H_2_O_2_), and hydroxyl radical (·OH). They have been recognized as crucial cell signaling molecules and regulate a wide variety of important cellular processes including cell survival, proliferation, differentiation, apoptosis, and DNA damage response [2]. It has been widely accepted that ROS are associated with cancer due to the fact that they may be able to induce transformation by causing DNA damage and that transformed cells have higher levels of ROS production than normal cells [3]. An increased production of ROS has also been associated with genomic instability and enhanced DNA damage including double strand breaks and also performs a signaling function to promote cell proliferation and migration, thus, contributing to leukemic cell transformation [4]. For example, in more than 60% of acute myeloid leukemia (AML) patients, ROS production is heavily increased due to NADPH oxidase (NOX) activation and strongly promotes AML proliferation [5]. Interestingly, protein tyrosine phosphatases (PTPs) are also among the various ROS targets. ROS inactivate PTPs transiently by oxidizing a reactive cysteine on the catalytic domain [6]. By doing so, ROS induce tyrosine phosphorylation and activation of many important signaling molecules including mitogen-activated protein kinase (MAPK), c-JUN N-terminal kinase (JNK), and p38 MAPK, therefore establishing themselves as important factors in growth factor signaling pathways [2,7].

**Figure 1:**
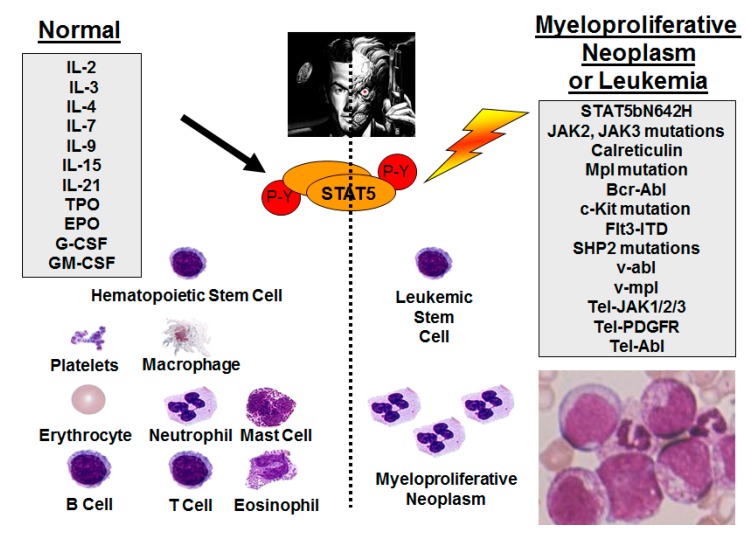
Signal transducer and activator of transcription-5 (STAT5) activation in normal and leukemic hematopoiesis—two faces of the same molecule. Different growth factors (left) normally activate STAT5 to drive hematopoietic differentiation in many lymphoid and myeloid lineages. In contrast, activating mutations (right), including some in STAT5b, result in persistent phosphorylation and transcriptional activation, which drives lymphoid and myeloid hematologic malignancies as well as myeloproliferative neoplasms.

ROS also contribute to tyrosine phosphorylation of signal transducer and activator of transcription (STAT) family members [8]. STATs carry out two functions: Transducing cellular signals and activating transcription of a diverse set of genes including those that are involved in cancer development. STATs are constitutively active in many solid tumors and hematologic malignancies due to enforced upstream kinase signals [9]. The Janus kinase-signal transducer and activator of transcription (JAK-STAT) pathway controls survival, proliferation, and differentiation in several cell types. STATs are also persistently active in a number of cancers, especially myeloproliferative neoplasms and leukemias [10]. Among the STAT proteins, STAT5 is encoded by two closely related genes STAT5a and STAT5b and plays a major role in regulating vital cellular functions such as proliferation, differentiation, and apoptosis of hematopoietic and immune cells [11,12]. STAT5 is activated by phosphorylation of a single tyrosine residue (Y694 in STAT5A and Y699 in STAT5B) and negatively regulated by dephosphorylation. A wide variety of growth factors and cytokines can activate STAT5 through the JAK-STAT pathway. The activation of STAT5 is transient and tightly regulated in normal cells but can become dysregulated in hematologic malignancies (Figure 1).

## 2. Persistent STAT5 Activation through Upstream Mutant Tyrosine Kinases

Persistent activation of STAT5 is mostly caused by activating mutations in upstream tyrosine kinases in a number of cancers. For example, in chronic myelogenous leukemia (CML), the Bcr-Abl translocation leads to expression of the Bcr-Abl fusion protein and constitutive activation of its tyrosine kinase activity. That causes aberrant activation of STAT5 [13]. By using a Bcr-Abl inhibitor, constitutive activation of STAT5 can be blocked [14]. In acute myeloid leukemia (AML), overexpression and constitutive activation of the FLT3 receptor tyrosine kinase induces a strong activation of STAT5, which can be inhibited by an FLT3 protein tyrosine kinase inhibitor [15]. Moreover, in mast cell (MC) neoplasms, the D816V-mutated variant of Kit promotes growth of hematopoietic progenitor cells and proliferation of neoplastic MCs through constitutive activation of the STAT5-PI3K signaling pathway [16]. Additionally, in chronic myelomonocytic leukemia, the TEL/PDGFβR tyrosine kinase fusion protein activates STAT5 [17].

JAK2 and JAK3 mutations can also persistently activate STAT5 by encoding constitutively active or hyperactive JAK proteins in many hematopoietic malignancies [10]. For instance, a gain-of-function JAK2 V617F somatic mutation constitutively activates the JAK-STAT pathway and is identified in the majority of patients with myeloproliferative neoplasm [18].

Apart from that, STAT5 can also be activated by members of the Src family kinases (SFKs) and in particular, c-Src through growth factor receptor signaling, which is different from the cytokine receptor signaling in the JAK pathway [19]. It has been shown that SFKs are frequently constitutively activated in leukemia cell lines and inhibitors of Src tyrosine kinases block constitutive activation of STAT5 in these cells [20].

## 3. STAT5b Gene Mutations in Leukemia/Lymphoma

Although there are relatively few cases of STAT5 mutation reported in human cancers and most of the aberrant activation of STAT5 is caused by mutations in upstream kinases, there are still some recent discoveries and identification of STAT5 mutations in patients leading to increased phosphorylation and activation of STAT5. For example, in patients with large granular lymphocytic (LGL) leukemia, two mutations: Y665F and N642H were identified, both located on exon 16 of STAT5b in the Src-like homology 2 (SH2) domain. The frequency of STAT5b mutation in all LGL leukemia patients is around 2%. However, no STAT5a mutations have been detected in LGL leukemia patients. From an in vitro analysis, both Y665F and N642H mutants increased tyrosine phosphorylation of STAT5b and also its transcriptional activity. Additionally, N642H mutant cells demonstrated a more prominent increase in STAT5b phosphorylation and transcriptional activity compared with Y665F. Accordingly, patients with the N642H mutation suffered from a more aggressive and fatal disease whereas typical LGL leukemia patients had a relatively favorable outcome [21,22]. Such activating mutations on STAT5b have also been observed in lymphomas derived from γδ-T or NK cells with a high frequency, especially in γδ-T-cell-derived lymphomas and enteropathy-associated T-cell lymphoma type II, where STAT5b mutations were observed in more than 30% of tumor samples [23]. The N642H mutation on STAT5b has also been identified as a driver mutation in both adult and pediatric acute lymphoblastic leukemia (T-ALL) prolymphocytic leukemia, NK/T cell lymphoma, and enteropathy-associated T cell lymphoma [24,25,26,27], whereas STAT5b T628S, Y665F, and V712E mutations have been discovered in multiple cases [24,27]. A few additional rare mutations in STAT5b such as E438K, G492C, P702A, I704L, and Q706L have been reported although the effects on STAT5b function are still unknown. In a transgenic mouse model, STAT5b^N642H^-expressing mice rapidly developed T cell and hepatosplenic T cell lymphoma but lacked a hematopoietic phenotype [28].

## 4. STAT5 Drives ROS Formation

Constitutive activation of STAT5 in cancer is associated with increased production of ROS through multiple mechanisms [29]. This has been proven by increased levels of cellular ROS upon enforced expression of STAT5 in BCR-ABL1 transformed cell lines by retrovirus transduction and further confirmed by decreased ROS production after the introduction of shRNA specific for STAT5 in K562 cells [30]. Such STAT5-mediated increase of ROS production has also been validated in vivo, where mice injected with transformed cells ectopically expressing STAT5 had a higher ROS level in the spleen compared with mice injected with transformed cells only expressing GFP [30].

STAT5 mediates ROS production through a mechanism independent of JAK2 or mitochondrial respiration [30]. In FLT3/ITD-positive AML cells, increased ROS levels appear to be produced through the direct interaction of tyrosine phosphorylated STAT5 with RAC1. RAC1 is a small GTPase protein and serves as an essential component of the NOX holoenzyme [31]. NOX is a family of proteins consisting of seven homologs and several subunits. They serve the major function of electron transportation through the plasma membrane and the generation of ROS including superoxide [32]. Inhibition of FLT3-ITD not only reduced tyrosine phosphorylation of STAT5 but also decreased RAC1 activity and its binding to NOX [29].

Another mechanism has also been proposed regarding increased ROS formation in FLT3/ITD-positive cells. NOX4 belongs to the NOX family proteins. It is expressed in many cell types including hematopoietic stem cells and its expression can be induced by the endoplasmic reticulum (ER) [32]. NOX4 is a direct downstream target of STAT5 since the NOX4 promoter possesses STAT binding elements and it has been found by ChIP assays that STAT5 binds to these elements in a FLT3/ITD-dependent manner. Therefore, NOX4 expression level is upregulated in FLT3/ITD-positive cells owing to the constitutive activation of STAT5 as an FLT3 downstream effector. NOX4 then contributes to ROS formation since knockdown of NOX4 by siRNA or shRNA significantly reduced cellular ROS levels [4,33]. Interestingly, either by inhibiting the FLT3 tyrosine kinase activity or by inhibiting the NOX activity, ROS formation in the ER was reduced. However, such effects were not observed for mitochondrial ROS, suggesting that FLT3-ITD drives endogenous ROS production in the ER through NOX proteins [34].

Apart from enhancing transcription of NOX proteins, oncogenic STAT5 signaling also promotes ROS formation by repressing expression of antioxidant enzymes including catalase and glutaredoxin-1 (Glrx1) in Bcr-Abl-positive CML. Both catalase and Glrx1 function to reduce the cellular ROS level. After introducing a Bcr-Abl inhibitor to Bcr-Abl-positive human leukemia cell lines, the expression level of catalase and Glrx1 increased while the activity of STAT5 decreased. Moreover, cell lines transformed with a constitutively active STAT5 mutant had reduced expression levels of catalase and Glrx2 compared with control cell lines [35]. It is important to note that Casetti reported that STAT5, and in particular STAT5A, can be protective against oxidative stress based on the observation that the knockdown of STAT5A increased the basal level ROS production and subsequently genomic stress in CML cell lines [36]. However, in this case, STAT5B was still present and had a differential contribution to stress protection.

## 5. ROS Promotes STAT5 Phosphorylation

**Figure 2:**
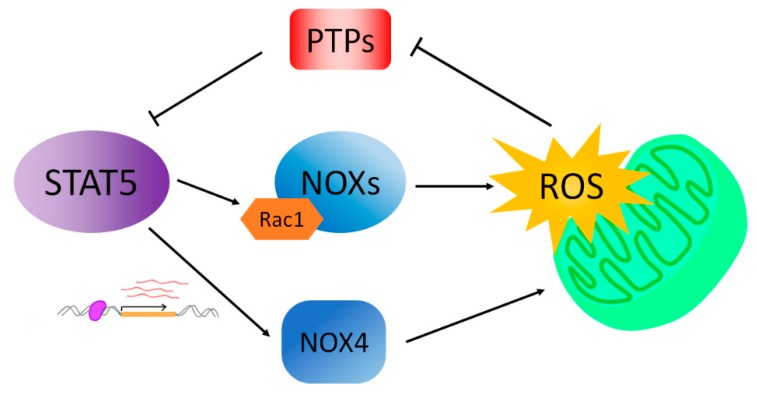
STAT5 cooperates with reactive oxygen species (ROS) in a feedforward and feedback manner. Close connections between STAT5 and ROS provide leverage for therapeutic targeting. Inhibition of ROS permits liberation of inhibitory phosphatases which remove the phosphate from activated STAT5 thus causing de-activation.

While STAT5 activation increases ROS production, ROS also play a role in the activation of STAT5 (Figure 2). By using the antioxidant N-acetyl-_L_-cysteine (NAC), which inhibits ROS production, Mitsuko was able to demonstrate that NAC significantly inhibits the IL-3 induced tyrosine phosphorylation of JAK2 kinase and furthermore, activation-specific phosphorylation of STAT5 [37]. The mechanism behind the ROS enhancement of the JAK2-STAT5 signaling pathway remains to be explored. However, there is increasing evidence suggesting that redox regulation of protein tyrosine phosphatases (PTP) may play a role in the ROS-mediated increase of tyrosine phosphorylation. PTPs oppose the activity of protein tyrosine kinases (PTK) by removing a phosphate group from tyrosine residues in target proteins. Therefore, the tyrosine phosphorylation level of cellular proteins is controlled by a coordination between PTKs and PTPs [38]. Interestingly, PTPs are intracellular targets of ROS. All PTPs contain a cysteine residue at their active sites. The cysteine residue can be oxidized by H_2_O_2_ to cysteine sulfenic acid, leading to the inactivation of PTP enzymatic activities. Such oxidation and inactivation can be reversed with thiol compounds [39] and the reversible inhibition of PTP activity contributes to the ligand-induced increase in receptor tyrosine kinase signaling [40]. Additionally, redox regulation of JAKs has been described [41,42].

## 6. Non-Canonical STAT5 Activation in High Risk Subsets of Acute Myeloid Leukemia

AML is a heterogeneous and complex disease with a low remission and high relapse rate [43]. Mutations in FLT3, NPM1, KIT, RAS, and CEBPα are found in 30–50% of AML patients [44,45,46]. JAK-STAT and PI3K-Akt-mTOR pathways are very important downstream mediators of survival and proliferation signals that are generated by these molecular mutations in AML. Persistently activated STAT5 is associated with poor prognosis [47,48] and is a bona fide therapeutic target [49]. The pSTAT5^high^ sub-type is associated with approximately half of all AML where it promotes leukemia stem cell (LSC) self-renewal [47,50] and resistance to tyrosine kinase-inhibitors [51,52]. Emerging new agents, such as the multi-kinase inhibitor midostaurin [53,54], advocate that a multi-faceted approach can succeed.

Interestingly, the mechanism of activation of STAT5 by mutant FLT3 and KIT is due to non-canonical signaling not observed in normal cells [16,55,56]. Endoplasmic reticulum (ER)-localized NOX4 combined with aberrant FLT3-ITD or KIT (D816V) N-linked glycosylation traps these receptor tyrosine kinases (RTKs) on the ER and permits reactive oxygen species (ROS) accumulation and activation of STAT5 independent of JAKs [35,57,58,59]. Therefore, there is potential for a favorable therapeutic index sparing normal hematopoietic cells with agents that are able to exploit mislocalized RTK signaling and target STAT5.

## 7. Emerging ROS-Reducing Cancer Therapies

The FLT3-ITD mutation is found in 20–30% of patients with AML and portends a poor prognosis; despite intensive chemotherapy and allogeneic stem cell transplantation. FLT3-ITD is also commonly found associated with MLL-fusions, which are also associated with poor outcomes. Recent strategies using targeted therapies (i.e., FLT3 tyrosine kinase inhibitors—TKIs), have thus far yielded modest responses and most relapsed/refractory patients will still die of their leukemia. STAT5 is critical for normal hematopoiesis [60,61,62,63]. Therefore, specificity for the inhibition of STAT5 only in leukemia but not normal cells is critical for the design of new anti-leukemia therapies that selectively target high risk pSTAT5^+^ sub-sets. STAT5 activation can be uncoupled from canonical JAK2-STAT5 activation, thus JAK2 inhibitors are not a good option for AML and have had limited clinical success as single agents. Class III RTKs include five immunoglobulin-like extracellular domains and include PDGFRα, PDGFRβ, c-KIT, c-FMS, and FLT3. Several of these RTKs are activated in AML and in particular the c-KIT and Flt3 mutant forms are considered poor risk. There is a therapeutic need for targeting mutant FLT3 and c-KIT driven AML by exploiting their unique ER-localization [64] and non-canonical activation of STAT5. RTK-driven leukemia cells are characterized by reprogrammed calcium homeostasis [65,66] that favors proliferation and survival.

**Figure 3:**
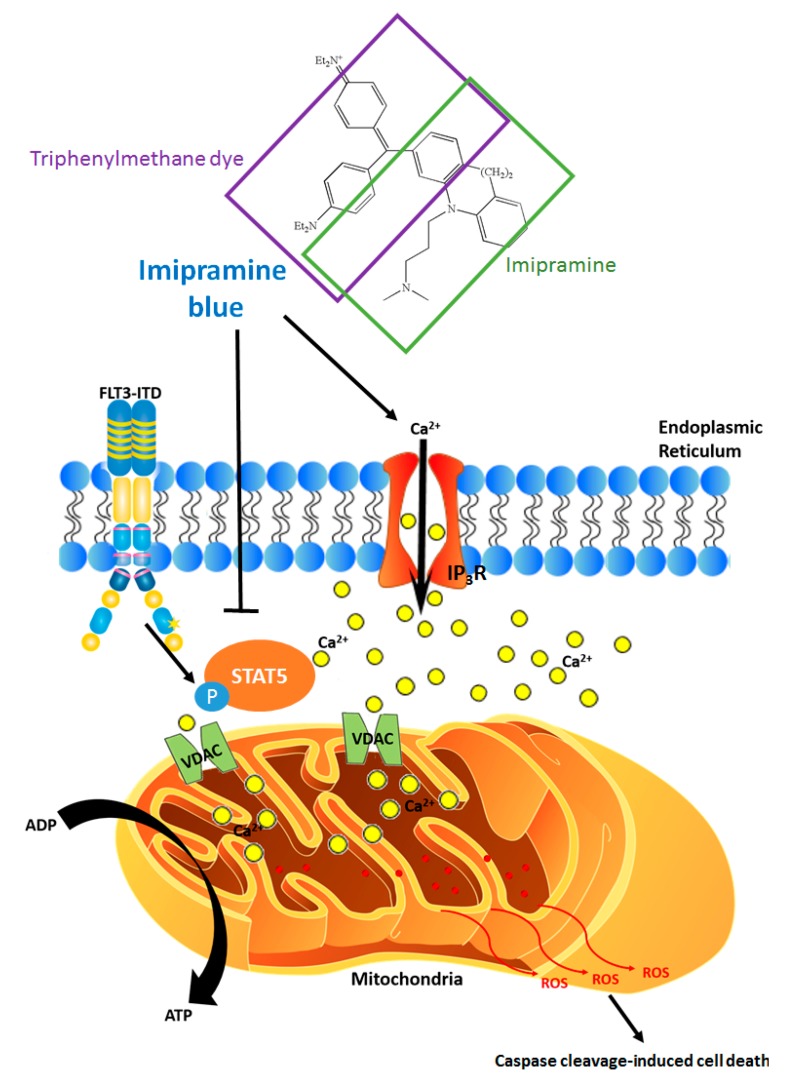
Non-canonical STAT5 activation in leukemia and therapeutic targeting using novel endoplasmic reticulum (ER)-targeted chimeric drugs. FLT3 mutants are defective in glycosylation and are trapped on the ER, near the surrounding areas of high local concentrations of ROS, which conveniently leads to favorable conditions for phosphorylation of STAT5. Drugs that are selectively trapped in the ER-lysosome compartments can suppress local ROS. An example is shown of Imipramine Blue, a chimeric drug that combines a triphenylmethane backbone (purple) with imipramine (green), an FDA approved anti-depressant. Agents with this potential are ideal candidates for leukemia selective indirect targeting of STAT5. Alternatively, drugs that induce calcium release from the ER or increase mitochondrial sensitivity to released calcium have the potential to induce mitochondrial outer membrane permeabilization and caspase cleavage-induced cell death.

Imipramine blue (IB) is a chimeric drug developed by Jack Arbiser at Emory University [67]. IB was generated by combining a triphenylmethane dye backbone [68] through reaction of Michler’s ketone, with imipramine, an FDA-approved tricyclic antidepressant. The initial rationale was to increase the lipophilicity of triphenylmethane dyes (e.g., gentian violet) by conjugating to a lipophilic amine (e.g., imipramine) to optimize uptake across the blood-brain barrier. The dye backbone quenches ROS and inhibits NADPH oxidase. Fortuitously, in addition to delivery to the brain for glioma therapy [67], lipophilic amines selectively accumulate in the ER [69] and lysosomes [70] and imipramine can activate phospholipase C [71,72]. We have found IB to be extremely effective and selective against subsets of AML expressing mutant FLT3 or c-KIT, including drug-resistance conferring FLT3 mutants [73]. The enhanced selectivity of IB may be related to the trapping of IB in the ER/lysosomes combined with the requirement for an ER-localized FLT3 mutant for effective tyrosine phosphorylation of STAT5 in AML (Figure 3).

## 8. ROS-Calcium Connection and Potential for ROS-Increasing Cancer Therapies

**Figure 4:**
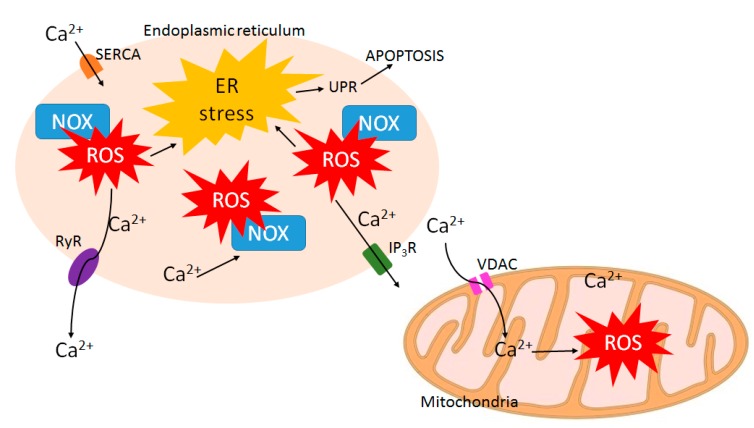
Calcium-ROS connection in controlling ER-mitochondria cross-talk. ER-resident NADPH oxidase (NOX) produce ROS, which induces calcium release and ER stress, leading to the unfolded protein response (UPR). This response can subsequently cause apoptosis. Calcium release also leads to mitochondrial ROS production and this second pathway can also lead to apoptosis through mitochondrial outer membrane permeabilization (MOMP). The mechanism of IB-induced cell death has previously been shown to involve calcium release from the ER, MOMP, and caspase-induced cell death. In contrast, TG-induced cell death involves calcium release via inhibition of the sarco(endo)plasmic reticulum calcium ATPase (SERCA) as well as potent induction of ER-stress and UPR. It will be important to understand how IB and TG differ mechanistically in regard to their anti-leukemic properties.

Calcium plays an important role in cell signaling as a second messenger and calcium signaling serves essential functions in many cellular processes including cell proliferation, gene transcription, and apoptosis [74,75]. Considering its importance in determining cell fate, calcium signaling is often altered and remodeled in cancer cells facilitating tumor progression [74]. Interestingly, ROS signaling and calcium signaling are closely related and share complex bidirectional interactions between each other [76] (Figure 4).

On one hand, an increase of the intracellular calcium level can induce ROS production by activating enzymes that are involved in ROS formation. Those enzymes include NOX, which can be stimulated by calcium-dependent proteins [77]. Calcium also increases ROS generation by stimulating the Krebs cycle and oxidative phosphorylation in mitochondria [78].

On the other hand, ROS can regulate calcium signaling by modulating a variety of calcium channels. The sarcoplasmic/endoplasmic reticulum (SR/ER) is the major site of calcium storage in eukaryotic cells [79]. The SR/ER membranes are embedded with calcium release channels such as ryanodine receptors (RyR) and inositol 1,4,5-trisphosphate receptors (IP_3_R). Both RyR and IP_3_R channel activities can be enhanced by ROS, either through direct oxidation or by regulation of NOX [76]. Therefore, ROS increase calcium release from the SR/ER and elevate the intracellular calcium level. Such calcium release from the ER is an indicator of ER stress. ER stress is caused by perturbation of ER homeostasis. Such condition activates the unfolded protein response (UPR) to re-establish homeostasis. However, although initially serving as a protective mechanism, the UPR is toxic to cells when it is prolonged and will eventually lead to mitochondrial apoptosis [80].

Therefore, increased Calcium-ROS has the potential for use as a drug therapy. Notably, in addition to the ROS quenching role of IB, it can also induce calcium release from the ER and drive cell death. Therefore, IB functions in a dosage-dependent manner, with lower doses effectively increasing intracellular calcium and higher doses suppressing ROS and STAT5 tyrosine phosphorylation. These differences might come into play in scenarios where either bulk leukemia or LSCs are being targeted since STAT5 is a major regulator of LSC self-renewal [50,81].

## 9. Exploiting the ROS-STAT5 Kinome to Identify New Therapeutic Targets

The STAT5 whole “kinome” that links ROS with STAT5 phosphorylation, as well as downstream ROS and calcium-dependent gene expression, may be exploited to identify potential therapeutic targets of value for high risk AML. One such starting point could be calcium-regulated kinases. The ER is the largest membrane-bound organelle in eukaryotic cells and is the largest calcium store. Calcium release is known to target leukemia cells [82] but specificity for RTK-driven AML has not been reported. Diverse anti-depressants are structurally lipophilic amines that cross the blood-brain barrier efficiently and modulate phospholipids in neurons [83,84]. They have also been implicated as chemosensitizers [85,86] and possibly could be chemopreventative [87]. Due to their lipophilic properties, some compounds can efficiently accumulate in the ER or mitochondria where they quench ROS and suppress STAT5 tyrosine phosphorylation. Commercially available ER-tracker and Mito-tracker dyes have been selected for these basic properties [69]. Calcium and ROS have an intimate and complex connection and many drug targets require careful study since a calcium overload could lead to side effects that are widely known for anti-depressant drugs.

Two important calcium-regulated genes, calcium/calmodulin-dependent protein kinase II (CAMKII) [88] and a nuclear factor of activated T-cells 1 (NFATc1) [89], are expressed in leukemia cells that are characterized by high levels of ROS. NFATc1 is a DNA-binding transcription factor causative for FLT3-ITD positive AML that is resistant to the kinase inhibitor sorafenib [89]. This function is believed to be mediated through its effects on driving Ras oncogene expression. CAMKII is believed to function through the regulation of critical signaling pathways involved in STAT3 transcriptional activation [88]. In contrast to the potential pro-leukemic roles of calcium, high risk AML may also be characterized by increased calcium storage, facilitating a “primed state” whereby perturbation of ER calcium storage might be especially effective at inducing apoptosis. In leukemia, the unfolded protein response (UPR) is a protective mechanism against ER stress that is regulated by XBP1 and IRE1. However, in presence of prolonged or severe ER stress, the pro-survival signal of the UPR turns into a toxic signal that induces mitochondrial apoptosis as shown in Figure 3 [80]. While XBP1 is expressed as a splice variant (XBP1s) in 16% of AML, the functional role of this splicing event is not well characterized. XBP1s is not associated with FLT3-ITD mutation and confers favorable prognosis [90]. Therefore, while unlikely that the UPR is involved in the initial drug sensitivity, it may be induced by IB or pimozide (PIM).

Calcium-release inducing therapeutic approaches hold promise for selectively targeting cancer cells. It was recently demonstrated that AML selective cytotoxicity in vivo could be achieved with the SERCA antagonist curcumin, a natural product found in turmeric and curry powders [82]. Similarly, it has been shown that thapsigargin (TG), the most potent SERCA antagonist could be effectively dosed in vivo in a mouse model of T-ALL. It was found through a complementary genetic screen that both SERCA and Notch [91] are targets of TG. Despite these advances in demonstrating the proof-of-principle for SERCA inhibition in cancer, there remain significant barriers to implementing this strategy and optimizing beyond pre-clinical studies. There are numerous natural products that are ER or SERCA targeted to be considered [92] for applications in AML but also myeloproliferative neoplasms driven by JAK2 and MPL mutants. However, because of the key role of calcium oscillations in muscle and brain function, these approaches come with significant risk for serious side effects. There has been some progress in this area in recent years and it might be able to overcome limitations through the use of novel drug delivery.

Calcium-inhibiting therapeutic approaches are characterized by pimozide (PIM), an FDA approved antipsychotic drug of the diphenylbutylpiperidine class and acts as an antagonist of the D2, D3, and D4 dopamine receptor and the 5-hydroxytryptamine receptor [93]. Pimozide is known to inhibit T-type voltage-gated calcium channels [94], which can promote cancer cell proliferation [95]. PIM was identified as a STAT5 inhibitor by a high throughput screen based on STAT transcriptional activity and it decreased the survival of CML cells resistant tyrosine kinase inhibitors [96]. PIM is able to have a combinatorial effect with TKI midostaurin and sunitinib in the inhibition of induction of apoptosis in FLT3-driven AML [97]. Recently, we showed that PIM inhibited pSTAT5 at a dose larger than 5 µM in the MV4-11 cell line. Additional, reduced expression of STAT5 target genes and selective induction of apoptosis was observed. Using a sub-optimal dose of IB and pimozide, this combination was highly synergistic and selective in FLT3/ITD^+^ cell lines, including those with FLT3 point mutations, with little effect on FLT3/ITD negative cell lines or on CD34^+^ cord blood cells. Furthermore, this combination was also selective and synergistic for 32D cells transduced with a c-KIT D814V mutant [73]. In contrast, no synergy has been observed between TG and pimozide (K.D.B., unpublished observations), suggesting that the mechanism of action of IB and TG may be unique.

## 10. STAT5 in Normal Hematopoiesis—Potential Side Effects of STAT5 Inhibition

STAT5 regulates normal lympho-myeloid development through activation downstream of early-acting cytokines, their receptors, and JAKs and plays a critical role in stem cell self-renewal; both in normal and leukemic stem cells. The greatest challenge to direct targeting of STAT5 signaling is to find the potential therapeutic window for leukemia cells while having minimal cytotoxicity to normal cells. STAT5 can be activated by the upstream tyrosine kinase. Therefore, TKIs could be initially effective to inhibit STAT5 activation but could have significant long-term off-target toxicity and the TKI resistance usually occurs with further increased activation. Recently, a few STAT5-specific inhibitors such as 13a [98], Stafib-2 [99], and AC-4-130 [100] have been reported to target the STAT5 SH2 domain. AC-4-130 treatment can inhibit proliferation and clonogenic growth in both AML cell lines and primary AML patient cells with IC_50_ between 1.6 to 4.9 µM but less toxicity against healthy CD34^+^ cells with IC_50_ between 6.3 to 7.6 µM [100]. It also shows synergistic treatment effects with TKI although whether the therapeutic window is wide enough to spare normal healthy donors still needs to be further investigated. As STAT5 activation promotes an overall elevation of ROS, targeting ROS could provide additional treatment avenues, especially in leukemia characterized by persistent STAT5 phosphorylation. ROS-targeting, in combination with TKI or STAT5 specific inhibitors, might further improve therapeutic benefits in leukemia. However, since ROS is closely aligned with mitochondrial function, the cellular phenotypes associated with STAT5 inhibition may need to be monitored closely. Autophagy is a protective mechanism that is induced when oxidative phosphorylation is inhibited and thus this could be a target for combination therapy.

## 11. Conclusions and Perspectives

It is an exciting time for molecularly targeted therapies directed at transcription factors, which make up prime targets for eradicating bulk leukemia and LSC populations. New agents have been described with in vitro and in vivo efficacy for direct and indirect targeting of STAT5. Indirect approaches rely on the liberation of inhibitory phosphatases or other negative regulators that normally comprise a negative feedback loop. Dysregulation of calcium-ROS in AML is also a vulnerability that remains to be fully exploited due to the difficulty of targeting these signaling intermediates without non-hematopoietic toxicity. Each approach presents its own strengths and weaknesses. The normal role of STAT5 in hematopoiesis has been extensively characterized during the past 20 years, showing virtually ubiquitous roles in all hematopoietic lineages as well as regulatory T-cells required to prevent severe autoimmunity. Due to this extensive connection of STAT5 in normal and leukemic hematopoiesis, innovative new approaches will be required for safe and effective long-term cures based on STAT5 inhibition. If STAT5 targeted therapies can be harnessed in a safe and targeted manner, they will be applicable for a wide range of pSTAT5^+^ leukemias as well as some solid tumors and thus have a significant potential for positive impact on future patient care.
